# Microtomographic, histomorphometric, and molecular features show a normal alveolar bone healing process in iNOS-deficient mice along a compensatory upregulation of eNOS and nNOS isoforms

**DOI:** 10.1590/1678-7757-2022-0436

**Published:** 2023-03-20

**Authors:** Carolina Fávaro FRANCISCONI, Priscila Maria COLAVITE, Angélica Cristina FONSECA, Michelle de Campos Soriani AZEVEDO, André Petenuci TABANEZ, Jéssica Lima MELCHIADES, Andreia Espíndola VIEIRA, Carlos Eduardo Palanch REPEKE, Marcela CLAUDINO, Gustavo Pompermaier GARLET

**Affiliations:** 1 Universidade de São Paulo Faculdade de Odontologia de Bauru Departamento de Ciências Biológicas Bauru SP Brasil Universidade de São Paulo, Faculdade de Odontologia de Bauru, Departamento de Ciências Biológicas, Bauru, SP, Brasil.; 2 Instituto de Ciências Biológicas e da Saúde Departamento de Histologia e Embriologia Maceió AL Brasil Instituto de Ciências Biológicas e da Saúde, Departamento de Histologia e Embriologia, Maceió, AL, Brasil.; 3 Universidade Federal de Sergipe Programa em Ciências Aplicadas Lagarto SE Brasil Universidade Federal de Sergipe (UFS), Programa em Ciências Aplicadas, Lagarto, SE, Brasil.; 4 Universidade Estadual de Ponta Grossa Departamento de Odontologia Ponta Grossa PR Brasil Universidade Estadual de Ponta Grossa (UEPG), Departamento de Odontologia, Ponta Grossa, PR, Brasil.

**Keywords:** Bone repair, Nitric oxide, Inflammation, Immune response, Cytokines

## Abstract

Inducible nitric oxide synthase (iNOS) is one of the enzymes responsible for the synthesis of nitric oxide (NO), which is an important signaling molecule with effects on blood vessels, leukocytes, and bone cells. However, the role of iNOS in alveolar bone healing remains unclear. This study investigated the role of iNOS in alveolar bone healing after tooth extraction in mice.

**Methodology:**

C57Bl/6 wild type (WT) and iNOS genetically deficient (iNOS-KO) mice were subjected to upper incision tooth extraction, and alveolar bone healing was evaluated by micro-computed tomography (μCT) and histological/histomorphometric, birefringence, and molecular methods.

**Results:**

The expression of iNOS had very low control conditions, whereas a significant increase is observed in healing sites of WT mice, where iNOS mRNA levels peak at 7d time point, followed by a relative decrease at 14d and 21d. Regarding bone healing, both WT and iNOS-KO groups showed the usual phases characterized by the presence of clots, granulation tissue development along the inflammatory cell infiltration, angiogenesis, proliferation of fibroblasts and extracellular matrix synthesis, bone neoformation, and remodeling. The overall micro-computed tomography and histomorphometric and birefringence analyses showed similar bone healing readouts when WT and iNOS-KO strains are compared. Likewise, Real-Time PCR array analysis shows an overall similar gene expression pattern (including bone formation, bone resorption, and inflammatory and immunological markers) in healing sites of WT and iNOS-KO mice. Moreover, molecular analysis shows that nNOS and eNOS were significantly upregulated in the iNOS-KO group, suggesting that other NOS isoforms could compensate the absence of iNOS.

**Conclusion:**

The absence of iNOS does not result in a significant modulation of bone healing readouts in iNOS-KO mice. The upregulation of nNOS and eNOS may compensate iNOS absence, explaining the similar bone healing outcome in WT and iNOS-KO strains.

## Introduction

Bone repair occurs throughout coordinated and interconnected stages, starting with a temporary inflammatory response, which constructively influences the following events.^1^ A low magnitude and transient production of inflammatory mediators in bone fracture sites supports osteogenic cellular differentiation and bone formation required for a positive healing outcome, being this concept supported by the cause-and-effect association between anti-inflammatory drugs and delayed bone healing.^2^

In this scenario, macrophages contribute to repairing and remodeling initially exerting pro-inflammatory effects associated with the M1 phenotype, followed by a phenotypic switch towards the dominance of the M2 type, which is anti-inflammatory and pro-reparative.^3 - 5^ Within the multiple and functional M1 markers, inducible nitric oxide synthase (iNOS, also called NOS2) stands out as a prototypic M1 product. iNOS comprises an inducible nitric oxide synthase isoform expressed in response to different stimuli usually described as the main factor responsible for triggering nitric oxide (NO) production in inflammatory environments.^6^

Nitric oxide (NO) is a small and highly diffusible reactive molecule, with many functions, including its role as a second messenger on several inflammatory events.^7 , 8^ Notably, although the production of NO can catalyze other nitric oxide synthases (NOS) isoforms, namely eNOS (endothelial NOS or NOS1) and nNOS (neuronal NOS or NOS3),^7 , 8^ iNOS is the main NO inducer along inflammatory immune responses. iNOS is induced in many cell types in response to several inflammatory cytokines (such as TNF and IL1b), hypoxia, and oxidative stress.^9^ These potential iNOS inducers are present in bone injury/healing sites,^7^ reinforcing the involvement of iNOS as part of the inflammatory process that precedes bone healing.

Notably, iNOS/NO axis influence in bone healing can extend its inflammatory properties. NO is described as a potent regulator of bone cells, since it can exert both anabolic and anti-resorptive effects in the bone, with both pre-clinical and clinical data supporting the role of NO in bone health.^10 , 11^ NO can inhibit the recruitment, proliferation, differentiation, activation, and/or survival of osteoclasts and their precursors *in vitro* .^12^ Accordingly, iNOS deficiency can accelerate osteoclast formation and bone resorption, decrease normal bone mass, and exacerbate bone destruction in arthritis models.^12 , 13^ Also, *in vitro* and *in vivo* evidences show NO in osteoblast differentiation and proliferation during bone development and healing.^11^

Thus, considering the involvement of iNOS in inflammatory responses and its modulatory effects on bone cells, it is reasonable to hypothesize iNOS involvement in alveolar bone healing process. Indeed, iNOS is described to be expressed during mandibular bone healing in rabbits, and deletion of iNOS gene impairs mouse fracture endochondral healing.^14^ However, despite the many *in vitro* studies, the few *in vivo* studies focused on the role of iNOS in bone repair remain to be complemented to properly address the real functions of iNOS in this process. In this context, this study investigated the role of iNOS intramembranous alveolar bone healing outcome, by the iNOS-KO and C57Bl/6-WT mice strain comparative analysis using micro-computed tomography (μCT) and histological, histomorphometric, immunological, and molecular methods.

## Methodology

### Animals

Experimental groups comprised 8-week-old male C57BL/6 wild-type (WT) and iNOS genetically deficient (iNOS-KO) mice (N=5 for micro-computed tomography and histological analyses; N=4 for the Real-Time PCR array analysis for each group/time point), maintained in the animal facilities of the Bauru School of Dentistry (FOB-USP). Throughout the study period, the mice were fed with sterile standard solid mice feed (Nuvital, Curitiba, PR, Brazil) and sterile water; except in the first 24 hours after surgery, in which the diet was crumbled. The experimental protocol was approved by the local Institutional Committee for Animal Care and Use (Committee on Animal Research and Ethics [Comissão de Ética no Ensino e Pesquisa em Animais] CEEPA-FOB/USP, processes No. 024/2011 and No. 011/2019) following the Guide for the Care and Use of Laboratory Animals.

### Experimental protocol and tooth extraction in mice model

Tooth extraction was performed as previously described,^1^ and overall subsequent analyses were performed following previously described protocols.^1 , 4^ The animals were anesthetized by intramuscular administration of ketamine chloride (Dopalen, Agribrans Brasil LTDA) and of xylazine chloride (Anasedan, Agribrands Brasil LTDA) and the right upper incisor was extracted. At the end of the experimental periods (zero hours and seven, 14, and 21 days post extraction), the animals were euthanized with an excessive dose of anesthetic, and the maxillae were collected. Five maxillae were subjected to micro-computed tomography (μCT) and histological analyses; and four samples containing only the region of the alveolus were subjected to the Real-Time PCR array analysis (the molecular analysis also comprised an additional independent group of WT iNOS-KO mice, not subjected to any surgical procedure, used to perform data normalization). Due to limited availability of knockout mice, the μCT and picrosirius analyses were only performed at the 7d and 14d time points. Samples for the μCT and histological analyses were fixed in PBS-buffered formalin (10%) solution (pH 7.2) for 48 hours at room temperature, then washed overnight in running water and maintained temporarily in alcohol fixative (70% hydrous ethanol) until the conclusion of the μCT analysis, decalcified in 4.13% EDTA (pH 7.2) and subjected to histological processing. Samples for molecular analysis were stored in RNAlater (Ambion, Austin, TX) solutions.

### Micro-computed tomography (μCT) evaluation

The maxillae samples were scanned as previously described^1^. Projection images were reconstructed using the NRecon software and three-dimensional images obtained by the CT-Vox software. Morphological parameters of trabecular bone microarchitecture were evaluated using the CTAn software according to the recommended guidelines.^15^ A cylindrical region of interest (ROI) with an axis length of 3mm (100 slices) and diameter of 1mm was determined by segmenting the trabecular bone located from the coronal to apical thirds. Tissue volume (TV), bone volume (BV), bone volume fraction (BV/TV, %), trabecular thickness (Tb.Th, mm), trabecular number (Tb.N, mm), and trabecular separation (Tb.Sp) were determined.

### Histomorphometric analysis

Histomorphometric analysis was performed as previously described^1^. Serial sections were obtained using a microtome (Leica RM2255, Germany) and stained with hematoxylin and eosin. Morphometric measurements were performed by a single calibrated investigator with a binocular light microscope (Olympus Optical Co., Tokyo, Japan) using a 100× immersion objective and a Zeiss kpl 8× eyepiece containing a Zeiss II integration grid (Carl Zeiss Jena GmbH, Jena, Germany) with 10 parallel lines and 100 points in a quadrangular area. In the morphometric analysis, points were counted coinciding with the images of the following components of the alveolar socket: clot, inflammatory cells, blood vessels, fibroblasts, collagen fibers, bone matrix, osteoblasts, osteoclasts, and other components (empty space left by the inflammatory exudate or intercellular liquid and bone marrow). The results were presented as the volume density for each evaluated structure.

### Picrosirius-polarization method and quantification of birefringent fibers

The Picrosirius-polarization method and quantification of birefringent fibers were performed to evaluate the structural changes in the newly formed trabecular bone matrix based on the birefringence of the collagen fiber bundles, as previously described.^1^ Serial sections with 5μm thickness were cut and stained with Picrosirius Red Stain. Picrosirius Red-stained sections were analyzed using a polarizing lens with a binocular inverted microscope (Leica DM IRB/E), and all images were captured with the same parameters. The region of interest was delimited by the Adobe Photoshop CS6 software, totalizing 1,447,680 pixels. The quantification of the intensity of birefringence brightness was performed using the AxioVision 4.8 software (CarlZeiss). For quantification, the images were binarized for definition of the green, yellow, and red color spectra, and the quantity of each color of pixels corresponding to the total area enclosed in the alveoli was measured. Mean values of four sections from each animal were estimated in pixels.^2^

### Real-Time PCR array reactions

Real-Time PCR array reactions were performed as previously described.^4^ The extraction of total RNA from the remaining alveolus was performed with the RNeasy FFPE kit (Qiagen Inc., Valencia, CA) according to the manufacturers’ instructions. The integrity of the RNA samples was verified by analyzing 1mg of total RNA in a 2100 Bioanalyzer (Agilent Technologies, Santa Clara, CA) according to the manufacturers’ instructions, and the complementary DNA was synthesized using 3μg of RNA by a reverse transcription reaction (Superscript III, Invitrogen Corporation, Carlsbad, CA). Real-Time PCR array was performed in a ViiA7 instrument (LifeTechnologies, Carlsbad, CA) using a custom panel containing the targets “Wound Healing” (PAMM-121), “Inflammatory cytokines and receptors” (PAMM-011), and “Osteogenesis” (PAMM-026) (SABiosciences, Frederick, MD), as well as for NOS isoforms (iNOS, nNOS, and eNOS), used for gene expression profiling. Real-Time PCR array data was analyzed by the RT2 profiler PCR Array Data Analysis online software (SABiosciences, Frederick, MD) to normalize the initial geometric mean of three constitutive genes (GAPDH, ACTB, Hprt1) and then normalized by the control group (comprising an additional independent group of mice, not subjected to any surgical procedure), and expressed as fold change regarding the control group, as previously described.^4^

### Statistical analysis and data availability

Differences among data sets were statistically analyzed by One-Way analysis of variance (ANOVA) followed by the Bonferroni multiple comparison post-test or Student’s *t* -test when applicable; the Mann-Whitney and Kruskal-Wallis (followed by Dunn’s test) tests were used for data that did not fit in the distribution of normality. The statistical significance of the experiment involving the PCR Array was evaluated using the Mann-Whitney test, and the values tested for correction with the Benjamini-Hochberg Procedure. Values of p<0.05 were considered statistically significant. All statistical tests were performed with the GraphPad Prism 5.0 software (GraphPad Software Inc., San Diego, CA). The datasets generated during and/or analyzed during this study are available from the corresponding author on reasonable request.

## Results

### Kinetics of iNOS expression along the bone healing process

**Figure 1:**
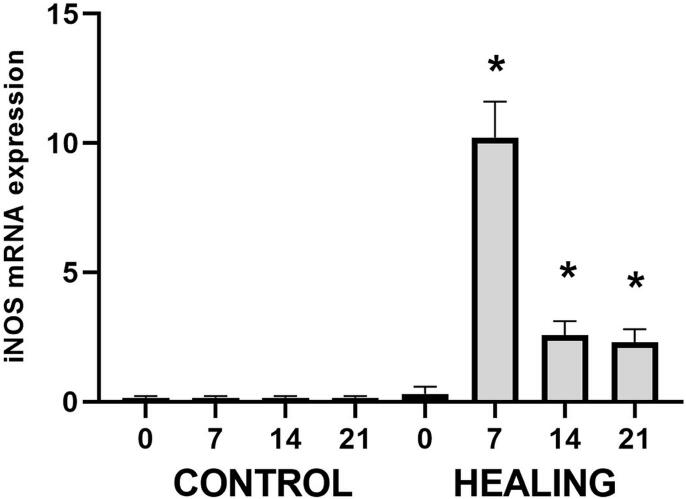
Kinetics of iNOS expression along the bone healing process and its correlation with M1 macrophages. Molecular analysis (Real-Time PCR Array) of iNOS mRNA expression kinetics along the bone healing process in C57Bl/6 mice. Results were obtained when comparing the relative expression of iNOS in the different groups normalized by Gapdh, Actb, and Hprt1; * represents the differences (p<0.05) between healing sites and the control group at each time point

First, we analyzed the kinetics of iNOS expression along the bone healing process. Our results ( Figure 1 ) show that iNOS expression in control conditions is very low at all time points; however, we observed a significant increase in healing sites at all time points. The iNOS mRNA levels peak at the 7d time point, followed by a decrease at 14d and 21d, when the expression remains higher than in control conditions.

### μCT analysis

**Figure 2:**
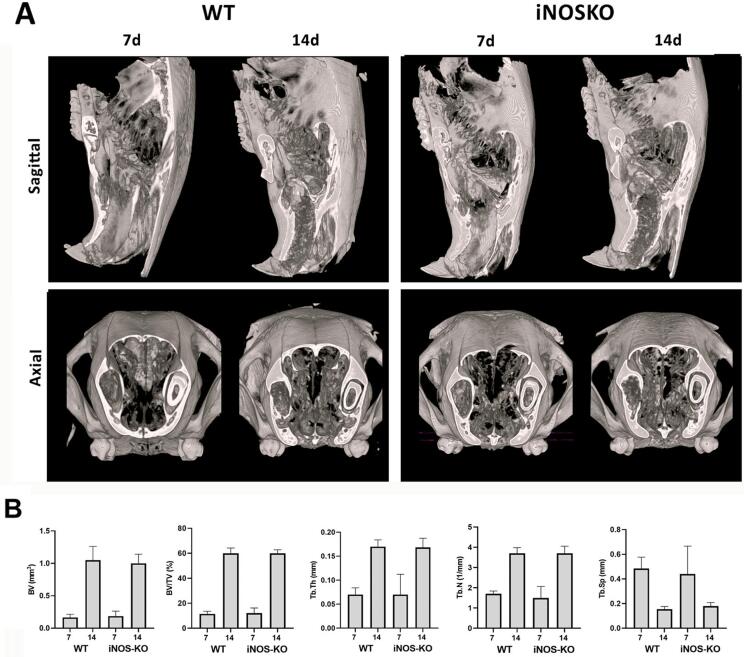
Micro-computed tomography (μCT) analysis of the kinetics of the bone healing process in C57Bl/6 WT and iNOS-KO mice. Samples from C57BL/6 wild-type (WT) and iNOS-KO mice were scanned with the μCT System (Skyscan 1174; Skyscan, Kontich, Belgium) at seven and 14 days post tooth extraction to evaluate the kinetics of the bone healing process. Images were reconstructed using the NRecon software and three-dimensional images obtained with the CT-Vox software. (A) Representative sections of the alveolar bone healing kinetics at seven and 14 days post extraction of the right upper incisor in WT mice in sagittal and axial views. (B) Morphological parameters of the trabecular bone microarchitecture were evaluated using the CTAn software from the cylindrical region of interest (ROI) determined by segmenting the trabecular bone located from the coronal to apical thirds. Trabecular measurements analyzed included: bone volume (BV), bone volume fraction (BV/TV, %), trabecular thickness (Tb.Th, mm), trabecular number (Tb.N, mm), and trabecular separation (Tb.Sp); * represents the differences (p<0.05) between iNOS and WT group at each time point

Three-dimensional images from the μCT of maxillae containing alveolar bone healing were compared between the groups at seven and 14 days after tooth extraction. Similar to a previous study^1^, hyperdense areas compatible with the beginning of bone formation were observed at seven days. At 14 days, hyperdense areas showed more advanced bone formation with the trabecular bone reaching the central region of the socket in both groups ( Figure 2 ). We confirmed the morphological observations derived from the μCT reconstruction upon quantitatively evaluating bone microarchitecture features ( Figure 2 ). We analyzed the total tissue volume (TV), bone volume (BV), bone volume fraction (BV/TV), trabecular thickness (Tb.Th), trabecular number (Tb.N), and trabecular separation (Tb.Sp) in WT and iNOS-KO mice. We observed that total tissue volume (TV), bone volume (BV), and bone volume fraction (BV/TV) progressively increased over the periods, as well as the trabecular thickness (Tb.Th) and trabecular number (Tb.N) in both groups ( Figures 2A, B ). Moreover, we observed a reduction in trabecular separation (Tb.Sp) in the 14-day period ( Figure 2B ). Comparing the groups, we found no statistical difference between them.

### Histological and Histomorphometric analyses

**Figure 3:**
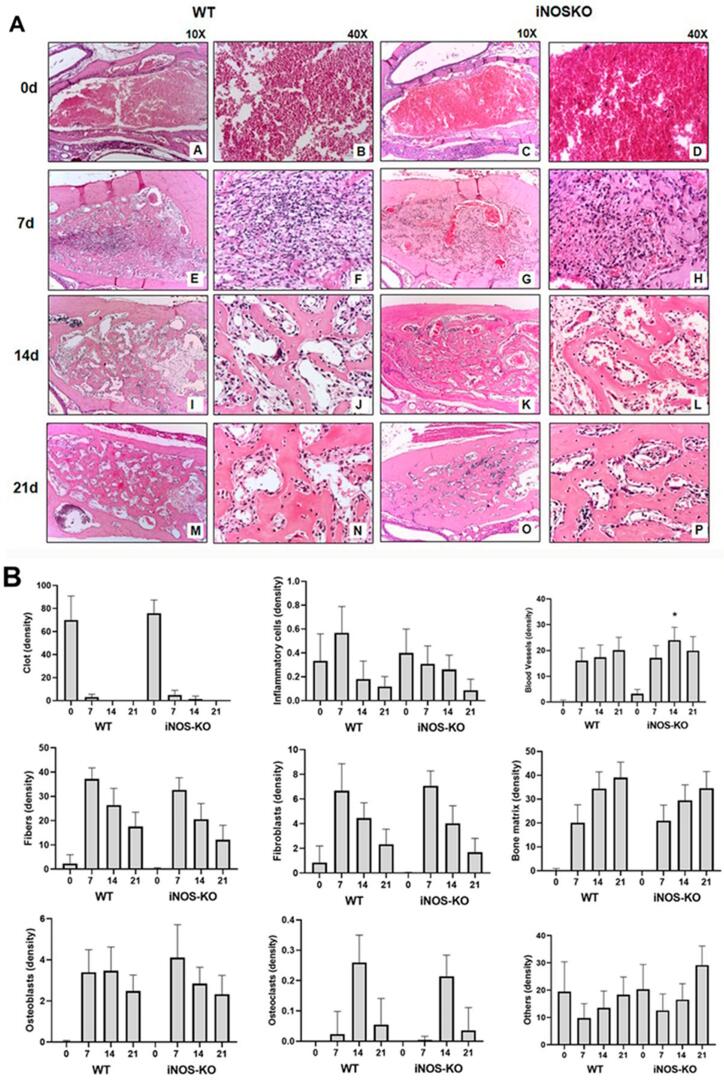
Histological and histomorphometric analysis of the alveolar bone healing analysis of the kinetics bone healing process in C57Bl/6 WT and iNOS-KO mice. Samples from C57BL/6 wild-type (WT) and iNOS-KO mice were subjected to histological and histomorphometric analyses at zero, seven, 14, and 21 days post tooth extraction to evaluate the kinetics of the bone healing process. (A) Photomicrographs representing the middle region of the dental alveolus in 10× and 40× magnifications. (B) Results presented as the means of density for each structure of the alveolar socket: collagen fibers, fibroblasts, blood vessels, inflammatory cells, clot, and other components (empty space left by intercellular liquid and spaces). Moreover, the results show the total density of connective tissue (represented by the sum of collagen fibers, fibroblasts, blood vessels, and inflammatory cells). The results represent the values of the mean and standard deviation in each of the periods analyzed; *represents the differences (p<0.05) between iNOS and WT group at each time point

The histological and histomorphometric analyses showed that the healing process occurs similarly in both groups, exhibiting sequential phases characterized by the presence of clot, granulation tissue formation with inflammatory cells infiltration, angiogenesis, proliferation of fibroblasts and collagen synthesis, bone formation, and remodeling ( Figure 3 ); this descriptive analysis is supported by the histomorphometric analysis of each parameter ( Figure 3 ). We observed a large amount of clot in WT mice at the initial period of the alveolar bone healing, then it decreased drastically ( Figure 3 ). At seven and 14 days, we observed the formation of connective tissue with a great number of collagen fibers, fibroblasts, and blood vessels ( Figure 3 ). We also observed a sequential decrease in the density of non-mineralized connective tissue, fibroblasts, and inflammatory cell volume density, parallel with new bone formation ( Figure 3 ). In the 21st day post extraction, the amounts of fibers and fibroblasts were smaller when compared with previous periods (seven and 14 days), showing the regression of soft tissue with the formation of bone tissue. We detected inflammatory infiltrate from the beginning of the bone healing process (0h), peaking in the period of seven days post extraction and then decreasing ( Figure 3 ). Compared to WT, iNOS-KO mice presented discrete differences in the alveolar bone healing process ( Figure 3 ). They also had a high clot concentration in the initial period of alveolar healing (0h). Then, we observed a high amount of collagen fibers and fibroblasts (seven and 14 days) and, finally, the presence of bone tissue (21 days) ( Figure 3 ). We detected inflammatory infiltrate from the beginning of the bone healing process, but this group presented a lower concentration of inflammatory cells in the seventh day post extraction when compared with the WT group ( Figure 3 ). Only in the 14th day post extraction we observed a statistically significant difference between the groups, in which iNOS-KO mice presented higher volume density of blood vessels, when compared with WT mice ( Figure 3 ; p<0.05). The osteoblast volume density had a significant increase at 7d and 14d, with a subsequent reduction at the 21d time period in both groups ( Figure 3 ). Osteoclasts peaked at 14d ( Figure 3 ) and were also observed in small numbers at the different periods evaluated. Finally, the histomorphometric analysis showed a higher quantified volume density of other structures in the final alveolar healing process, probably due the presence of bone marrow ( Figure 3 ), with no difference between groups.

### Matrix birefringence analyses

**Figure 4:**
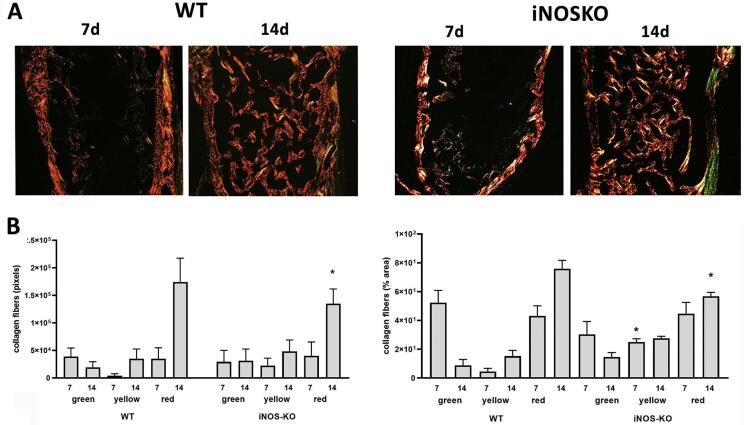
Birefringence analysis of the alveolar bone healing analysis of the kinetics of the bone healing process in C57Bl/6 WT and iNOS-KO mice. Samples from C57BL/6 wild-type (WT) and iNOS-KO mice were subjected to birefringence at seven and 14 days post tooth extraction to evaluate the kinetics of the bone healing process. (A) Photomicrographs representing the middle region of the dental alveolus, captured under polarized light, showing the deposition of collagen fibers, Stain Picrosirius Red; 10× objective. (B) Periods of seven and 14 days represented regarding the area in pixels of birefringent fibers relative to the total area, and the area of birefringent green, yellow, and red fibers throughout the experimental periods. The results represent the values of the mean and standard deviation in each of the periods analyzed; * represents the differences (p<0.05) between iNOS and WT group at each time point

At 7d, the presence of granulation tissue was characterized by many newly formed small blood vessels, an intense inflammatory cell infiltrate, and immature connective tissue with many fibroblasts and immature collagen fiber bundles ( Figure 4A ). At this time, fiber emitting birefringence in green tones (immature fibers) ( Figure 4A ) was found in abundance. At 14d, the maturation of connective tissue was evidenced by the presence of mature collagen fiber bundles ( Figure 4B ), when about three quarters of the fibers presented the red color spectrum, representative of a mature matrix ( Figure 4B ). Comparing the percentage of fibers between different strains, the iNOS-KO mice had a higher amount of immature (green) fibers at 14d compared to the WT mice (p<0.05) ( Figure 4B ). Similarly, yellow fibers were significantly more abundant in iNOS-KO mice than WT mice during all experimental periods (p<0.05) ( Figure 4B ), whereas mature fibers (red) were significantly more abundant in WT mice (p<0.05) ( Figure 4B ).

### Kinetics analysis of gene expression patterns in the bone healing process

**Figure 5:**
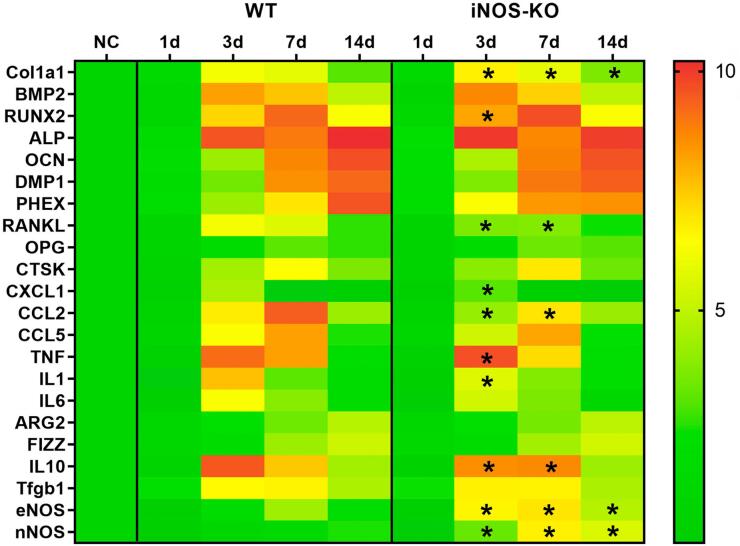
Molecular analysis of the alveolar bone healing analysis of the kinetics of the bone healing process in C57Bl/6 WT and iNOS-KO mice. Samples from C57BL/6 wild-type (WT) and iNOS-KO mice were subjected to Real-Time PCR array analysis at zero, seven, 14, and 21 days post tooth extraction to evaluate the kinetics of gene expression of the bone healing process. Molecular analysis (Real-Time PCR array) using a heat map to quantify the expression of the ECM and bone formation markers, osteoclast markers, inflammatory cytokines, M1 and M2 markers, and iNOS isoforms eNOS and nNOS were evaluated. Results are expressed as fold change relative to a normalizing control (C, i.e., normal alveolar bone) sample and normalized by three constitutive genes (GAPDH, ACTB, Hprt1); * represents the differences (p<0.05) between iNOS and WT group at each time point

Gene expression analysis by Real-Time PCR array showed an overall similarity between iNOS-KO and WT mice. The expression of ECM and bone formation markers, osteoclasts markers, chemokines, cytokines, and M1 and M2 markers in WT mice replicates the patterns described in previous studies.^1 , 4 , 16 , 17^ Regarding gene expression patterns in healing sites of iNOS-KO animals, some punctual modulations were observed, specifically in RANKL (upregulated at 7d and 14d time points), CXCL1 (upregulated at 7d), CCL2 (upregulated at 7d and 14d), IL-10 (upregulated at 7d and 14d), TNF and IL-1 (both upregulated at 7d). Regarding minor effects of iNOS deficiency in the outcome of alveolar bone healing, the expression patters of the other NOS isoforms, namely nNOS and eNOS, was evaluated despite the significant increase in iNOS expression along the healing process ( Figure 5 ). The molecular analysis showed that in WT mice both nNOS and eNOS had an upregulation along the healing process. Moreover, in the absence of iNOS, we observed an increased expression of eNOS in the periods of seven and 14 days when compared with WT animals, whereas nNOS isoform presented an upregulated expression in the periods of 14 and 21 days post extraction when compared with WT mice.

## Discussion

A low magnitude and transient inflammatory response in bone fracture sites is associated with a positive healing outcome, a concept supported by delayed bone healing upon the administration of anti-inflammatory drugs. Within the host inflammatory immune response mediators, iNOS is a potential modulator of the host response and bone healing outcome. Therefore, by taking advantage of an experimental model of the intramembranous bone healing process after tooth extraction in mice^1^, we investigated the role of iNOS in the bone healing process by comparing and WT and iNOS knockout mice strains by microtomographic, histomorphometric, and molecular investigations.

Initially, our results showed that iNOS expression is significantly upregulated in alveolar bone healing sites. Accordingly, previous studies describe the upregulation of iNOS in similar contexts, such as in bone fracture sites^13^. When iNOS expression profile is compared with the migration pattern of M1 cells in healing sites,^1 , 4 , 16 , 17^ we can observe a similar kinetics pattern between iNOS levels and CD80+ cells, reinforcing the possible iNOS/M1 interconnection along the bone healing process. Potential iNOS inducers, such as pro-inflammatory cytokines TNF, IL1B and IL6, and M1-markers,^1 , 4 , 16 - 18^ were expressed in bone healing sites with profiles resembling that observed in iNOS, also strengthening the putative involvement of iNOS as part of the inflammatory process that precedes bone healing.

However, the comparison of bone healing parameters in WT and iNOS-KO showed an overall similarity of healing kinetics and outcome in both strains. We observed no differences between the iNOS-KO and WT strains by the three-dimensional images from the μCT of maxillae, which had similar bone microarchitecture features upon quantitative evaluation of μCT reconstruction. Notably, bone healing in both the iNOS-KO and WT strains was similar to that previously described in the WT C57Bl/6 strain.^1 , 4 , 16 , 17^ Since only mineralized tissue features are subjected to the μCT analysis, we performed a histological and histomorphometric comparative analysis of healing sites. Nevertheless, similarly to the μCT analysis, the overall histological and histomorphometric features showed a similarity between bone healing features in the iNOS-KO and WT strains. Bone healing in the iNOS-KO strain involves initial blood clot formation, which is gradually replaced by a granulation tissue along leukocyte infiltration, in parallel with the proliferation of fibroblasts and endothelial cells, summarizing previous histological descriptions of early bone healing features.^1 , 17 , 19^ Among all the bone and healing parameters evaluated, the only significant differences observed between the WT and iNOS-KO strains are increased blood vessels in iNOS-KO animals at the 14d time point and a significant increase in collagen immature fibers compared to iNOS-KO at early times.

Regarding angiogenesis, previous studies show NO release at the injury sites, and results are associated with improved angiogenesis along the healing process.^20^ Accordingly, elevated levels of iNOS-derived NO release at the injury sites and are associated with angiogenic response in skin.^21^ Physical exercise-induced M2 polarization was associated with improved angiogenesis along the healing process, which is parallel with the decrease in iNOS+ M1 cells.^22^ Moreover, CD226 depletion results in decreased iNOS+ M1 counts and increased reparative collagen deposition and angiogenesis after myocardial infarction^23^. Besides enhanced angiogenesis, improved collagen deposition and maturation are induced by NO release at the injury sites.^20^ Modulation of collagen dynamics along the healing process are also described in the skin, in which iNOS downregulation mediated by COX-2 inhibition results in improved wound healing.^24^ Due to the divergent reports of iNOS modulation with angiogenesis and collagen neoformation in healing sites, we must consider the complexity of healing milieu, in which multiple cell types and mediators can be modulated in parallel with iNOS expression. Thus, caution is necessary when trying to address the role of iNOS based on scenarios in which its modulation involves broader changes in the microenvironment, such as the inhibition of inflammatory pathways of macrophage polarization dynamics.

We should also consider that, although iNOS is an important molecule upregulated during wound repair, its alleged contribution to a favorable healing outcome relies on the production of nitric oxide (NO). Although the determination of local NO production in alveolar bone healing sites is unfeasible due to technical reasons, we can argue that other NOS isoforms could be responsible for sustaining the local NO production in the absence of iNOS, explaining the similar healing course of iNOS-KO and WT animals, as well as the contradicting aforementioned results. The molecular analysis showed that both nNOS and eNOS isoforms were upregulated in the healing sites of iNOS-KO animals when compared with WT controls. Accordingly, previous studies report the expression of different NOS isoforms after femoral fractures in rats.^25^ The kinetics of NOS isoforms in alveolar bone healing sites resemble that described in femoral fractures, having an initial peak of iNOS, followed by sequential eNOS and nNOS peaks.^25^ Notably, our results show that in the absence of iNOS, eNOS presents an early upregulation, replacing the iNOS peak at 7d, and being sustained at high levels until 14d, when the nNOS expression is also significantly upregulated. Thus, considering that NO levels would ultimately comprise the final NOS pathway mediator involved in the healing process,^13^ we can hypothesize that regardless of the NO-inducing factor, the increased expression of eNOS and nNOS may compensate for the lack of iNOS, and this compensation would maintain the NO levels needed for the repair. Further studies specifically designed to individually address NOS isoforms are required to provide definitive evidence of the possible compensatory role of eNOS and nNOS.

In accordance with such hypothesis, considering the other M1 markers that accompany iNOS as the effectors of pro-inflammatory macrophages, our results show that the overall expression of TNFa, IL-1B, and IL-6 is similar in the iNOS-KO and WT strains. We observed a slight, but significant decrease in TNFa and IL-1B levels in the iNOS-KO strain at 7d, the same time point when iNOS expression peaks along the repair in the WT strain. At this same time point, we also observed a slight, but significant increase in the anti-inflammatory mediator IL-10. Since IL-10 is also regarded as a M2 product, a possible M1/M2 unbalance could be inferred. However, the levels of prototypic M2 markers ARG2 and FIZZ are similar in both strains, suggesting that, despite some fluctuation in pro- and anti-inflammatory mediators’ production at a given/single time point, the general M1/M2 balance is sustained along the healing process in iNOS-KO mice. Accordingly, a study showed that M1 polarization and effector response can occur even in the absence of iNOS, and that iNOS deficiency does not compromise M2 macrophage polarization and activation.^26^

Regarding bone markers, the molecular analysis supports the results of the microtomographic and histomorphometric analyses, since the evaluated markers expression in iNOS-KO mice resembles the WT counterparts. Specifically, the levels of bone-related growth factors (BMP2), transcription factors (RUNX2), activity markers (ALP, OCN), and maturation markers (DMP1, PHEX) are similar in iNOS-KO and WT strains along the healing process. Although the literature has many examples of the possible modulatory action of iNOS over osteoblasts, the previously discussed probable compensatory action of eNOS and nNOS over NO generation in healing sites may overcome iNOS deficiency, resulting in normal bone formation along the healing process. Notably, although the levels of osteoclastogenic mediator RANKL are significantly higher in the iNOS-KO strain at 7d and 14d, we observed no significant modulation of osteoclasts counts or alleged activity (based on the CTSK expression). Thus, similarly to the osteoblasts, despite the numerous studies describing possible modulatory activities of iNOS over osteoclasts^27 , 28^, the upregulated eNOS/nNOS levels may explain the overall maintenance of osteoclasts generation/activity in iNOS-KO mice.

Notably, our results clearly differ from the role of iNOS in chronic inflammatory responses associated with alveolar bone healing. In a model of periapical lesion, iNOS-deficient mice expressed increased IL-1β, TNFα, RANK, RANKL and MCP-1 levels when compared with WT mice and the progression of periapical lesion in these animals was related to an imbalance of proinflammatory cytokines (IL-1β and TNFα), bone resorption modulators (RANK and RANKL), and MCP-1^29^. Moreover, the iNOS-KO mice developed increased inflammatory cell recruitment and osteolytic lesions when compared with WT mice, associated with increased expression RANK and reduced expression of OPG.^30^ Thus, the nature and extent of the host inflammatory immune response can also influence the possible role of iNOS in the distinct/opposing environment (i.e., chronically exacerbated against transient controlled responses). And an additional hypothesis is that differential iNOS levels in such a distinct environment can influence the distinct outcomes, since the expression of iNOS in chronic inflammatory osteolytic lesions is significantly higher than in healing sites (data not shown). The effects of NO on bone metabolism are highly dependent on the amounts available, considering that in small quantities NO can favor bone formation and remodeling.^31^ In fact, effects of NO on bone function depend on its concentration,^32^ and low physiological levels of NO have a stimulatory effect on normal bone formation,^10^ development,^11^ remodeling,^33^ and fracture healing. However, the level of NO has inhibitory effects on all these processes, characterizing a pathological role/outcome.^34^ High concentrations of NO inhibit the activity and proliferation of osteoblasts and increases osteoclast activity in pathophysiological conditions.^7 , 8 , 12^ Moreover, the effects of NO on pre-osteoblasts and osteocytes are dose-dependent and bi-phasic *in vitro* : although low NO concentrations increase proliferation, differentiation and survival, high concentrations result in contrary effects.^35 - 37^The nature of iNOS-inducing stimuli also must be considered, since LPS (and other bacterial products) are the main host response trigger in osteolytic infectious lesions, whereas the release of damage-associated molecular patterns (DAMPs) can comprise the host response trigger in non-infectious conditions, such as in alveolar bone healing.

Furthermore, our results, i.e., similar healing process in iNOS-KO and WT strains, contradict previous reports of impaired bone healing in the absence of iNOS.^13 , 14^ However, such previous reports comprise the analysis of fracture healing, which involves endochondral bone formation, whereas alveolar bone healing comprises an intramembranous bone healing situation/scenario.^1^ The nature of cells surrounding the alveolar and “regular” (i.e., non-alveolar) bone clearly differs, being muscle stem cells, playing an essential role in fracture healing absent in alveolar bone healing.^38^ Moreover, alveolar bone healing typically occurs without histological cartilage formation, whereas endochondral ossification is a feature of long bone healing.^38^ Additionally, although bone fracture sites are normally regarded as a sterile milieu, the typical microbial challenge inherent to oral tissues surrounding the alveolar bone can modulate the bone healing outcome in the oral cavity.^1^

In short, our results show that although the expression of iNOS had very low control conditions, we observed a significant increase in healing sites of WT mice. The WT and iNOS-KO groups showed the usual and similar stages of bone healing, as well as an overall similar gene expression pattern in healing sites. This similar healing outcome possibly mediates a significant upregulation of nNOS and eNOS in the iNOS-KO group, suggesting that other NOS isoforms could compensate the absence of iNOS.

## Conclusion

The absence of iNOS does not result in a significant modulation of bone healing readouts in iNOS-KO mice. The upregulation of nNOS and eNOS may compensate iNOS absence, explaining the similar bone healing outcome in WT and iNOS-KO strains.
